# New Appliances and Things Medical

**Published:** 1911-01-07

**Authors:** 


### NEW APPLIANCES & THINGS MEDICAL.

**[We shall be glad to receive at our Office, 28 & 29 Southampton Street, Strand, London, W.C., from the manufacturers, specimens of all new preparations and appliances.]**

### PY-SHAU POINTS TEA.

(Thomas Christy and Co., Old Swan Lane, E.C.)

This tea consists of the points of young leaves, and contains no stalk or coarse leaf. It has been demonstrated by various investigators, notably by Kellner, that the amount of caffeine diminishes in leaves with their growth, so that young leaves contain more caffeine than old. It therefore follows that "Py-Shau Points Tea" contains a larger proportion of caffeine than does ordinary tea; it is further claimed that it contains a minimum of tannin, and in consequence is more suitable than the usual kinds for those affected by disorders of the digestive system. The sample submitted yields an infusion of pleasant taste and agreeable odour which corroborates the results of experiments recorded by E. Perrot and A. Goris, two workers who have conducted a considerable amount of research in connection with tea. The price is 2s. per lb.

### A NEW HEAD-REST.

(Hospitals and General Contracts Co., Ltd. 33 and 35 Mortimer Street, W.)

This head-rest is one of the simplest and most effective that we have seen. It consists of a silk cushion pillow attached to an elastic web sling fixed to the mahogany rest, which is tied to the bed by means of buckled straps. It is easy to apply to any bedstead, and is most comfortable, the elastic webbing giving it a spring which is greatly appreciated by the user. Its simplicity and strength will commend it to hospital and institutional workers, who will find it a durable and handsome piece of bed furniture, while it will be quite as much appreciated by private users. It is cheap and effective and thoroughly worth a trial.

### NEAVE'S FOOD.

(J. R. Neave and Co., Manufacturers, Fordingbridge.)

There are so many patent foods for infants and invalids on the market, and these are so extensively advertised that it is no easy matter to judge the merits of any one kind impartially. The samples of this food, which is already well known, were supplied to us in tins, priced 1s. 3d. each, each tin holding sufficient to make several pints of food. The tin labelled Neave's Milk Food contained a yellowish-white powder, holding a relatively large percentage of albuminoids and fats, with a very small amount of ash. The food is prepared by stirring a certain amount of the powder in hot water and boiling the mixture, when a slightly glutinous, pleasant-tasting fluid or gruel is obtained, which will be appreciated by those who have once tried it. Personally we confess that we have found it superior, in taste at least, to many other foods on the market. This food is designed for infants and is specially suitable for young children owing to the relatively high percentage of fat it contains. For invalids and as a food for adults, however, the second food, "Neave's Health Diet," is excellent, and prepared as a medium for cocoa it forms a pleasant and highly nourishing food, easily digested and assimilated. It contains about the same amount of albuminoids with a large amount of insoluble carbohydrate under relatively small fat percentage; it may be added to soups, puddings, stews, and various other dishes, and considerably improves these. As a convenient, easily prepared, and fairly concentrated food it is one of the best we have seen.

### SAL HEPATICA.

(Bristol Myers Company, Manufacturing Chemists, 277-281 Greene Avenue, Brooklyn, New York.)

Sal Hepatica contains laxative salts—mainly sulphate of soda—combined with lithium and sodium phosphate. It is an active antilithic and cathartic when used in ordinary doses: in small doses it forms an agreeable purgative, dissolved either in hot or cold water. Such a solution approaches very closely to the bitter water of such well-known spas as Marienbad and Püllna, and as a substitute for these it can be cordially recommended. The powder, which is non-effervescent, is put up in neat glass phials, and is free from any deleterious impurity or admixture. Sal hepatica is well worth a trial.

### A NEW BINAURAL STETHOSCOPE.

(Allen and Hanbury's, Ltd., 48 Wigmore Street, W.)

The instrument designed by Dr. Hayden Brown in 1908 has now been very extensively used and has become highly popular. Its advantages are undoubted, for it is simple in construction, light and portable (2 oz.), comfortable to use, easily adjusted, being kept in position by a spring which passes round the back of the head, getting rid of the cumbrous springs, clamps, and metal tubing of the older form. In use there is nothing to impede the vision between doctor and patient. At intervals during examination of patient—or during hospital bed-to-bed visitations—the stethoscope may be moved from the ears to hang round the neck; this is a great convenience and a practicable advantage in busy work. The instrument occupies the least possible space, and will go into the waistcoat pocket, owing to the smallness of its metal parts. A further advantage is found in the fact that the ear-pieces do not rotate in the ears in use, as is the case when the old form is moved in the least. The price is 7s. 6d.

### HORLICK'S MOTORING MAPS.

(Horlick's Malted Milk Co., Slough, Bucks.)

We have received copies of the motor maps for England and Wales, Scotland and Ireland, published by this firm, and we can thoroughly recommend them to the attention of medical and other motorists. The scale, 12 miles to the inch, is sufficiently ample for all practical purposes, and the maps are beautifully printed and may be thoroughly relied upon. They indicate definitely all first and second class motoring roads, all other roads, and all steamship routes, while they give, in a simple but ingenious manner, the distances between the various towns. With a tin of Horlick's Milk and copies of the three maps, the medical motorist ought to be able to visit any centre in the kingdom where a car can find roads to run on.

